# On-Demand Channel Bonding in Heterogeneous WLANs: A Multi-Agent Deep Reinforcement Learning Approach

**DOI:** 10.3390/s20102789

**Published:** 2020-05-14

**Authors:** Hang Qi, Hao Huang, Zhiqun Hu, Xiangming Wen, Zhaoming Lu

**Affiliations:** 1 School of Information and Communication Engineering, Beijing University of Posts and Telecommunication, Beijing 100876, China; qh.sirius@gmail.com (H.Q.); huanghao@bupt.edu.cn (H.H.); xiangmw@bupt.edu.cn (X.W.); 2 Beijing Key Laboratory of Network System Architecture and Convergence, Beijing University of Posts and Telecommunications, Beijing 100876, China; 3 Beijing Laboratory of Advanced Information Networks, Beijing University of Posts and Telecommunications, Beijing 100876, China; 4 School of Computing and Information Engineering, Hubei University, Wuhan 430062, China; zhiqunhu520@163.com

**Keywords:** WLAN, 802.11, channel bonding, multi-agent deep reinforcement learning

## Abstract

In order to meet the ever-increasing traffic demand of Wireless Local Area Networks (WLANs), channel bonding is introduced in IEEE 802.11 standards. Although channel bonding effectively increases the transmission rate, the wider channel reduces the number of non-overlapping channels and is more susceptible to interference. Meanwhile, the traffic load differs from one access point (AP) to another and changes significantly depending on the time of day. Therefore, the primary channel and channel bonding bandwidth should be carefully selected to meet traffic demand and guarantee the performance gain. In this paper, we proposed an On-Demand Channel Bonding (O-DCB) algorithm based on Deep Reinforcement Learning (DRL) for heterogeneous WLANs to reduce transmission delay, where the APs have different channel bonding capabilities. In this problem, the state space is continuous and the action space is discrete. However, the size of action space increases exponentially with the number of APs by using single-agent DRL, which severely affects the learning rate. To accelerate learning, Multi-Agent Deep Deterministic Policy Gradient (MADDPG) is used to train O-DCB. Real traffic traces collected from a campus WLAN are used to train and test O-DCB. Simulation results reveal that the proposed algorithm has good convergence and lower delay than other algorithms.

## 1. Introduction

Nowadays, IEEE 802.11 based Wireless Local Area Networks (WLANs) have been widely deployed around the world due to their low-cost and convenience. More and more users take WLAN as their first choice to access the Internet, which results in a fast growth of traffic. In order to meet the ever-increasing traffic, channel bonding is introduced in IEEE 802.11 standards. Channel bonding was first introduced in the IEEE 802.11n standard [1] where 802.11n nodes can transmit data packets in 40 MHz channel by bonding two contiguous non-overlapping 20 MHz basic channels. Then, the 802.11ac standard [2] further extends this capability by allowing the use of 80 MHz and 160 MHz channels by bonding four and eight basic channels, respectively. 802.11ax [3], which is the next generation WLAN standard, is expected to continue to develop channel bonding, such as supporting wider channels and non-contiguous channel aggregation (CA) [4]. At present, the heterogeneous WLANs where nodes have different channel bonding capability are very common.

**Figure 1:**
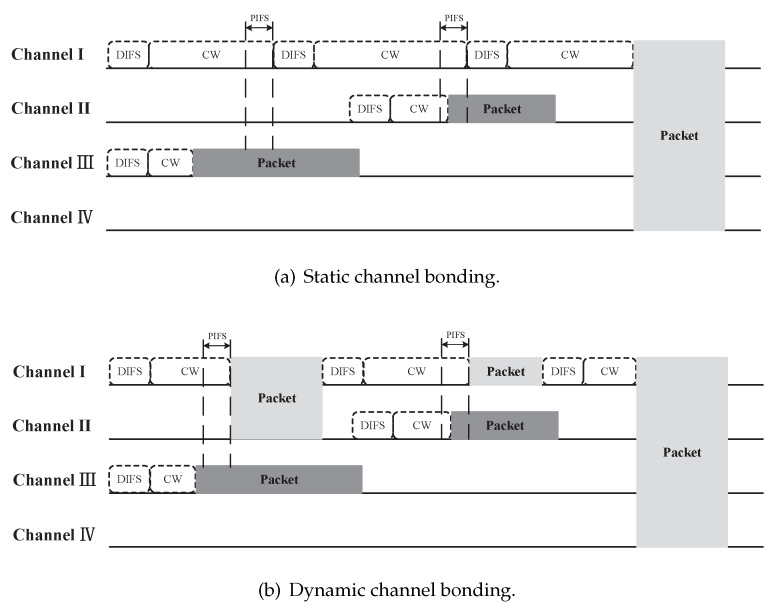
SCB and DCB in 802.11 standards.

**Figure 2:**
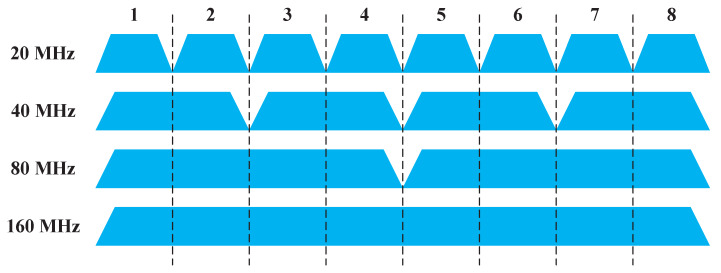
The channelization in 802.11 standards.

802.11 standards introduce two channel bonding schemes, static channel bonding (SCB) and dynamic channel bonding (DCB). The former is mandatory and the latter is optional. The access procedure of SCB and DCB is shown in Figure 1. At the beginning, a node determines its channel bonding parameter (P,B), where *P* is the index of primary channel and *B* denotes the number of bonded channels. A 20 MHz basic channel is selected as primary channel, and other basic channels are secondary channels. In the example of Figure 1, the channel bonding parameter is (I,4). The legacy Carrier Sense Multiple Access with Collision Avoidance (CSMA/CA) is performed on the primary channel. Specifically, after a Distributed Coordination Function (DCF) Inter-Frame Spacing (DIFS) period, the node samples a backoff time from contention window (CW) and waits for the backoff time to decrease to zero. Before the backoff time reaches zero, the node senses secondary channels for one Point Coordination Function (PCF) Inter-Frame Spacing (PIFS) period. If all the primary and secondary channels are idle, the node will start a transmission. The above procedure is same for SCB and DCB. The difference between SCB and DCB lies in the bonding choice when not all secondary channels are idle in PIFS. If the node uses SCB, it will give up transmission, reselect the backoff time and proceed previous procedure until all secondary channels are both idle. However, if the node uses DCB, it will bond idle secondary channels which are contiguous to the primary channel as many as possible, and start a transmission. Of course, the bonded channel should be consistent with the channelization regulated by 802.11 standards, as shown in Figure 2.

The pros and cons of channel bonding are both prominent [5,6,7,8,9]. On the one hand, larger channel bandwidth effectively increases transmission rate, which can achieve a lower delay. On the other hand, a wider channel will cover a bigger frequency scope, and this wider channel tends to increase the channel overlapping probability, which may cause severe performance degradation due to packet collision and overlength backoff time. The experiments and theoretical analyses in [6,10] show that the channel bonding parameter (P,B) has an important effect on the performance of channel bonding and should be selected carefully to guarantee the performance gains.

In fact, the traffic load differs from one WLAN access point (AP) to another. Furthermore, the traffic load changes significantly depending on the time of day [11,12,13]. For example, the APs located in a conference room have heavy traffic load only at meeting time and light load at other times, and the APs in offices usually have high load at working time. This raises a question of how to dynamically select channel bonding parameters for each AP to meet different and varying traffic demands and guarantee the performance gains.

According to the above analyses, in this paper, we proposed an On-Demand Channel Bonding (O-DCB) algorithm based on Deep Reinforcement Learning (DRL) [14] to select channel bonding parameter for heterogeneous WLANs, where the APs have different channel bonding capability. Selecting DRL is based on the following two reasons. The first reason is that DRL can learn the best policy by real-time interaction with the environment and only very minimal prior knowledge is needed. DRL embraces the learning ability of Reinforcement Learning (RL) and the generalization capability and approximation capacity of the Deep Neural Network (DNN). By online learning, it can effectively exploit the traffic pattern and adapt to the varying environment. Up to now, DRL and DNN have been applied in wireless communication to solve various problems [15], e.g., handoff management [16], base station sleeping [17] and performance prediction [18]. The second reason is that the periodicity and mobility of user behavior make the traffic loads exhibit some temporal and spatial correlations. According to the research findings in WLANs [12,17,19], the traffic load fluctuation shows repetitive pattern in weekday or week. DRL can catch this pattern and utilize it effectively.

To the best of our knowledge, this is the first work designing an on-demand channel bonding algorithm based on DRL. Simulation results reveal that the proposed algorithm has better performance than other channel bonding algorithms in terms of transmission delay. The main contributions of this paper can be summarized as follows:

- The on-demand channel bonding algorithm is designed in heterogeneous WLANs to decrease transmission delay, where APs have different channel bonding capability.
- The feasibility of DRL in channel bonding is explored in this paper. Real traffic traces collected from a campus WLAN [17] are used to train and test O-DCB, and simulation results show that O-DCB has lower delay than other channel bonding algorithms.
- In this problem, the state space is continuous and the action space is discrete. However, the size of action space increases exponentially with the number of APs by using single-agent DRL, which severely affects the learning rate. To accelerate learning, Multi-Agent Deep Deterministic Policy Gradient (MADDPG) [20] is used to train O-DCB.

The remainder of this paper is organized as follows. Section 2 presents related works, and Section 3 presents system model and problem definition. The preliminaries of RL is introduced in Section 4. Section 5 gives a detailed description of O-DCB. In Section 6, the convergence and performance of the proposed algorithm are verified by numerical results. Finally, Section 7 concludes the paper.

## 2. Related Works

### 2.1. Channel Bonding

The performance of channel bonding is studied in [5,6,7,8,9] via plenty of experiments and simulations, and the conclusions can be summarized as follows: (1) channel bonding can effectively increase throughput but is more susceptible to interference; (2) DCB usually has a better performance than SCB due to its flexibility, especially in node-intensive scenarios; (3) the primary channel and channel bonding bandwidth have important effects on the performance of channel bonding, and should be selected carefully to guarantee the performance gains.

On the other hand, a lot of works are devoted to analyzing the performance of channel bonding in theory. Bellalta et al. study the performance analysis of channel bonding, including SCB and DCB, in [10,21] by the use of Continuous Time Markov Network (CTMN), where the packet collisions are omitted and the traffic is saturated. Further research of channel bonding in high density WLANs, where nodes are not required to be within the carrier sense range of each other, are presented in [22]. Non-saturated traffic load scenarios are considered in [23,24,25] presents an analytical framework of Opportunistic Channel Bonding (OCB) where multiple channels shared by both legacy nodes and 802.11ac nodes with channel bonding capability. In [4], a renewal theory based analytical model is developed to study the performance of the DCB in 802.11ac, and non-contiguous CA in 802.11ax, with coexisting legacy single channel nodes.

Meanwhile, there are a number of papers which aim to improve channel bonding. In [26], the authors propose a MAC protection mechanism which effectively combats the hidden node problem on secondary channels. Stelter designs Channel Width Selection Scheme (CWSS) [27] which adjusts channel bandwidth according to data frame size, so as to improve bandwidth utilization. In [28], a channel allocation algorithm to achieve maximal throughput in DCB WLANs is proposed. Specifically, the throughput maximization is modeled as an integer nonlinear programming (INLP) problem and an optimal channel allocation algorithm based on the Branch-and-Bound Method (BBM) is used to solve the INLP. In [29], a reinforcement learning based channel bonding adaptation algorithm termed PoBA is developed to solve the hidden channel problem in 802.11ac networks. Quality of Service (QoS) requirements are considered in [30] and an enhanced dynamic channel bonding combining with the Transmission Opportunity (TXOP) [31] is developed. A primary channel selection scheme for 802.11ac nodes coexisting with legacy nodes to maximize network throughput is proposed and verified in [32]. Recently, a dynamic channel bonding algorithm [13] is proposed for 802.11ac WLANs, which considers the traffic demands of each WLAN. However, the authors do not consider the heterogeneity of nodes.

### 2.2. Spectrum Assignment

Other works concerning channel bonding in WLANs focus on spectrum assignment. Spectrum assignment is usually modeled as a graph coloring problem (an NP-complete problem) or non-convex optimization problem, and is solved by heuristic algorithm [33,34,35,36].

The works which likewise consider traffic demand and use spectrum assignment to solve include [11,37,38,39]. In [38], an on-demand channel bandwidth adaptation algorithm, SampleWidth, is proposed, which is designed for a single isolated link. Ref. [11] develops a load-aware adaptive channel bandwidth architecture for WLANs to improve spectrum utilization and per-load fairness. Further research of [11] termed FLUID is presented in [37]. However, these works assume that the demand of each AP is fixed, and the spectrum division is not standard-compatible. The scenario with uncertain demand is studied in [39]. The authors propose an adaptive channel bonding algorithm where center frequency and channel bandwidth are jointly allocated. However, the probability distribution of each AP demand is needed to know in advance, which is difficult to implement in practice. In the same way, above works both do not consider the heterogeneity of nodes.

## 3. System Model and Problem Definition

### 3.1. System Model

In this paper, we consider a wireless network consisting of *N* WLANs, V={1,2,⋯,N}, and a centralized controller (The proposed algorithm is especially suitable for enterprise and campus WLANs). Downlink transmission is considered in this paper. The type of WLANs include 802.11n, 802.11ac and 802.11ax. The centralized controller can control APs to adjust their channel bonding parameter and collect the traffic load information (e.g., load size, traffic arrival rate) from each AP. Without loss of generality, the time is discretized into isometric time slots $\left\{t_{1},t_{2},\cdots ,t_{j},\cdots ,t_{end}\right\}$ with the slot duration of δ. In each time slot, the channel bonding parameter of each WLAN remains unchanged. Because the number of users in each WLAN is unrelated to the research in this paper, for simplicity, we assume that WLAN *i* consists of an AP *i* and an user *i*.

**Figure 3:**
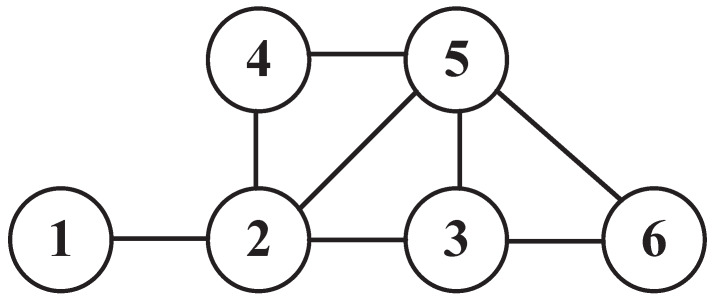
Conflict graph *G*.

We assume that the distance between AP and its user is short and the packet transmission failure is only caused by packet collision. As shown in Figure 3, a conflict graph G=(V,E) which is an undirected graph is used to represent the interference relationship between WLANs, where E is the set of edges. If there is an edge $e_{i,i^{\prime }}\in E$ and the channels of WLAN *i* and WLAN $i^{\prime }$ are overlapping, there may be packet collision between them. ${\bar{V}}_{i}=\left\{v|e_{v,i}\in E,v\in V\right\}$ denotes all the WLANs adjacent to WLAN *i* in *G*. For example, in Figure 3, ${\bar{V}}_{1}=\left\{2\right\}$ and ${\bar{V}}_{2}=\left\{1,3,4,5\right\}$.

There are *K* basic channels with the size of 20 MHz where the total channel bandwidth is 20K MHz, and the channelization which is regulated by 802.11 standards is shown in Figure 2. The set of optional channel bonding parameters for AP *i*, $C_{i}$, is determined by the AP type and *K*. For example, when K=4, there are 8 channel bonding parameters for 802.11n APs, i.e., $C_{i}=\left\{\left(1,1\right),\left(2,1\right),\left(3,1\right),\left(4,1\right),\left(1,2\right),\left(2,2\right),\left(3,2\right),\left(4,2\right)\right\}$, and there are 12 channel bonding parameters for 802.11ac/ax APs, i.e., $C_{i}=\left\{\left(1,1\right),\left(2,1\right),\left(3,1\right),\left(4,1\right),\left(1,2\right),\left(2,2\right),\left(3,2\right),\left(4,2\right),\left(1,4\right),\left(2,4\right),\left(3,4\right),\left(4,4\right)\right\}$. It is assumed that all other parameters affecting the transmission rate (e.g., transmit power, modulation and coding scheme, the number of spatial streams and guard interval) are fixed except for the channel bandwidth, which is denoted as $R_{B}$.

### 3.2. Problem Definition

Consider a wireless network contains multiple WLANs with different channel bonding capability. There may be interference between WLANs and each AP has varying traffic demand. Our objective is to find the optimal channel bonding parameters for all APs with the consideration of channel bonding capability, varying traffic demand and potential interference relationships, in order to decrease the average transmission delay of the total network. The delay is defined as the time between the packet arriving at the AP buffer and the packet being received correctly.

As is mentioned above, this problem is a complicated sequential decision problem and traditional optimization algorithms are unsuitable. Fortunately, the research findings in [12,17,19] show that the traffic load fluctuation in WLANs shows repetitive pattern in weekday or week, which is caused by the periodicity and mobility of user behavior. On the other hand, DRL can catch the pattern and find the optimal channel bonding parameters by interacting with the environment. As a result, we design O-DCB algorithm based on DRL. Before presenting O-DCB in detail, the preliminaries on RL will be briefly introduced in the next section.

## 4. Preliminaries on RL

This section gives a brief description of RL. For a comprehensive presentation, please refer to [14,40,41].

**Figure 4:**
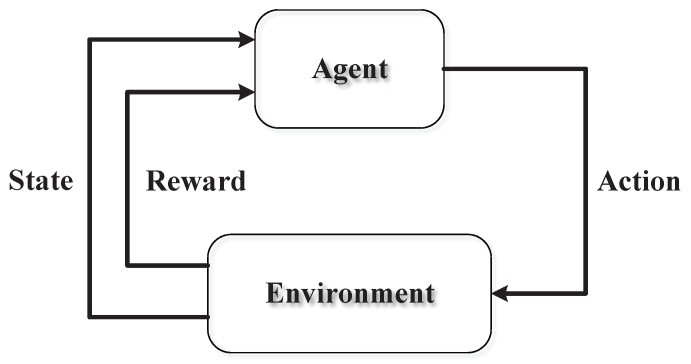
The basic component and form of reinforcement learning.

RL is learning how to map state to action, so as to maximize a numerical reward. The learner, i.e., the agent, is not told which actions to take, but instead must discover which actions yield the most reward by trying them, as shown in Figure 4. Markov Decision Processes (MDPs) are a mathematically idealized form of the RL problem [14]. An MDP is defined by a 4-tuple (S,A,P,r), where S is a finite set of states, A is a finite set of actions, *P* is a transition probability from state *s* to state $s^{\prime }$ after the action *a* is executed, and *r* is the immediate reward received after transitioning from state *s* to state $s^{\prime }$ due to action *a*. The goal of an MDP is to find a policy π:S→A to maximize the accumulated reward. The expected accumulated reward starting in state *s* and following policy π, termed state-value function, is defined as

(1) $$v_{\pi }\left(s\right)=E_{\pi }\left[\sum_{k=0}^{\infty }\gamma^{k}r_{t+k}|S_{t}=s\right],$$

where $\gamma \in \left[0,1\right]$ is the discount factor. Therefore, the optimal policy $\pi^{*}$ can be defined as

(2) $$\pi^{*}=\underset{\pi }{\mathrm{argmax}}\;v_{\pi }\left(s\right).$$

Similarly, the expected accumulated reward of taking action *a* in state *s* under a policy π is defined as

(3) $$Q\left(s,a\right)=E_{\pi }\left[\sum_{k=0}^{\infty }\gamma^{k}r_{t+k}|S_{t}=s,A_{t}=a\right],$$

which is called action-value function or *Q*-function. The optimal action-value function $Q^{*}\left(s,a\right)=\max_{\pi }Q_{\pi }\left(s,a\right)$ can be used to choose the optimal actions. With $Q^{*}$, for any state *s*, the agent can simply find any action that maximizes $Q^{*}\left(s,a\right)$. That is,

(4) $$\pi^{*}\left(s\right)=\underset{a}{\mathrm{argmax}}\;Q^{*}\left(s,a\right).$$

Based on Equation (4), the most effective and widely used method to find $\pi^{*}$ without the need of the environment model is *Q*-learning [42]. In *Q*-learning, the action-value function is obtained by iterative processes, and the update rule is defined by    

(5) $$Q\left(s,a\right)\leftarrow Q\left(s,a\right)+\alpha \left[r+\gamma \max_{a^{\prime }}Q\left(s^{\prime },a^{\prime }\right)-Q\left(s,a\right)\right],$$

where α denotes learning rate and usually gradually decreases as the iteration. The core idea in *Q*-learning is to find the Temporal-Difference (TD) error between predicted value and current value, i.e., $r+\gamma \max_{a^{\prime }}Q\left(s^{\prime },a^{\prime }\right)-Q\left(s,a\right)$. Watkins et al. proved that *Q*-learning can converge to the optimal action-value function $Q^{*}$ with probability one [42].

*Q*-learning can efficiently obtain an optimal policy when the state space and action space are small. However, it is not applicable to the complex problems which have continuous and high dimensional state spaces. In order to overcome this shortcoming, the function approximator should be introduced to process large state spaces. Deep *Q*-network (DQN), which uses a DNN to represent the action-value function in *Q*-learning, can learn the optimal policy in continuous and high dimensional state spaces. In DQN, the action-value function can be denoted as Q(s,a∣θ), where θ are the weights of the DNN. θ are updated to reduce the mean-squared TD error L(θ) by the use of stochastic gradient descent, and L(θ) can be denoted as

(6) $$L\left(\theta \right)=E\left[{\left(r+\gamma \max_{a^{\prime }}Q\left(s^{\prime },a^{\prime }|\theta^{-}\right)-Q\left(s,a|\theta \right)\right)}^{2}\right],$$

where $\theta^{-}$ are the DNN weights in previous iteration.

Because RL is unstable or even to diverge when a nonlinear function approximator such as a neural network is used [43], experience replay and target *Q*-network are used to guarantee the convergence. In experience replay, a replay memory is used to store the agent’s experiences $\left(s,a,r,s^{\prime }\right)$. If the replay memory is full, the oldest experiences will be discarded. The DNN is updated by sampling a minibatch randomly from the replay memory. As a result, the correlations between experiences are broken, and the variance of learning is reduced. In target *Q*-network, a copy of the DNN is created and updated periodically. It is used to generate the target values as shown in Equation (6), which further improving the stability of learning.

Further, to deal with the complex tasks which have both continuous, high dimensional state spaces and action spaces, Deep Deterministic Policy Gradient (DDPG) [41] is introduced. DDPG is a model-free off-policy actor-critic DRL algorithm. In particular, the DDPG algorithm maintains a parameterized actor neural network $\mu (s|\theta^{\mu })$ which specifies the current policy by deterministically mapping states to a specific action. The parameterized critic neural network $Q(s,a|\theta^{Q})$ is used to approximate the action-value function. The actor network is updated by applying the chain rule to the expected return from the start distribution *J* with respect to the actor parameters as follows:

(7) $$\begin{array}{cc} \nabla_{\theta^{\mu }}J & \approx E\left[\nabla_{\theta^{\mu }}Q\left(s,\mu \left(s|\theta^{\mu }\right)|\theta^{Q}\right)\right]\\ \; & =E\left[\nabla_{a}Q\left(s,\mu \left(s\right)|\theta^{Q}\right)\nabla_{\theta^{\mu }}\mu \left(s|\theta^{\mu }\right)\right]. \end{array}$$

Similarly, experience replay and target network are used in DDPG to guarantee the convergence. Not the same as DQN, the weights of target networks are updated by slowly tracking the original networks: $\theta^{\prime }\leftarrow \tau \theta +\left(1-\tau \right)\theta^{\prime }$ with the tracking parameter τ≪1.

## 5. On-Demand Channel Bonding Algorithm

In this section, we design an on-demand channel bonding algorithm based on DRL. However, single-agent DRL is not suited to this problem due to the vast action space. In particular, in single-agent DRL, the agent should select channel bonding parameters for all APs, therefore the action is a *N*-dimensional vector where the *i*-th element is the channel bonding parameter of AP *i*. As a result, the size of action space equals $\prod_{i=1}^{N}\left|C_{i}\right|$ and increases exponentially with *N*, where $\left|\cdot \right|$ denotes the cardinality of a set. The vast action space severely affects the learning rate and even makes the algorithm unachievable in practice. On the contrary, multi-agent DRL has good scalability. In multi-agent DRL, the size of action space is $\left|C_{i}\right|$ for agent *i*. As such, we use a multi-agent DRL algorithm, MADDPG [20], to accelerate learning.

MADDPG is an extension of DDPG in multi-agent settings. It adopts the framework of centralized training with decentralized execution where the critic network is augmented with extra information about the policies of other agents, while the actor network only has access to local information. The extra information is only used to ease training and not used during the test. In MADDPG, each AP corresponds to an agent, and the basic three elements (state, action and reward) for agent *i* are designed as follows.

### 5.1. State

The state—more accurately, the observation—of agent *i* in time slot $t_{j}$ is defined as

(8) $$s_{i,j}=\left\{l_{i,j},\lambda_{i,j-1}\right\},$$

where $l_{i,j}$ denotes the load size of AP *i* at the beginning of $t_{j}$, and $\lambda_{i,j-1}$ is the average load arrival rate in the last time slot.

### 5.2. Action

Obviously, the action $a_{i,j}$ is

(9) $$a_{i,j}=\left(P,B\right),$$

where $\left(P,B\right)\in C_{i}$. $C_{i}$ is determined by the type of AP *i* and the number of basic channels, and may be different for different APs.

### 5.3. Reward

Because our objective is to reduce delay of the total network, the reward is designed as

(10) $$r_{i,j}=\eta \cdot {\bar{d}}_{j}+\epsilon \cdot {\bar{l}}_{j+1},$$

where ${\bar{d}}_{j}$ is the average transmission delay of the total network in $t_{j}$. ${\bar{l}}_{j+1}$ is the average load size of all APs at the end (start) of $t_{j}$ ($t_{j+1}$), and ${\bar{l}}_{j+1}$ = $\frac{\sum_{i}l_{i,j+1}}{N}$. η and ϵ are both weighting factor and less than zero.

### 5.4. O-DCB

The pseudo-code of O-DCB is presented in Algorithm 1. First of all, the actor network μ and the critic network *Q* are initialized for each agent, and the corresponding target networks are built. A replay buffer with a fixed size $S_{RB}$ is also built. It should be noted that the replay buffer is shared by all agents but their actor-critic networks are personal.

At the start of each episode, the state of each agent is initialized to {0,0} because there are not any packet in each AP. At the start of time slot $t_{j}$, agents obtain the actions by their current actor networks $\mu_{i}$, as shown in line 4. To support discrete actions in this problem, the Gumbel-Softmax estimator [44] is used. The output of actor network is the probability mass function (PMF) of all actions, and the action is determined by sampling from this PMF. Then, the APs change their channel bonding parameters according to the actions and the new parameters remain unchanged until the end of $t_{j}$. Next, the rewards and new states are obtained, and the global state $s_{j}$, global action $a_{j}$, global reward $r_{j}$ and new global state $s_{j+1}$ in this time slot are stored in the replay buffer. Next, for each agent *i*, it samples a minibatch with a size of *L* from the replay buffer. The agent calculates loss using the target networks of all agents, the samples in the replay buffer and its critic network, as shown in lines 11–12. The critic network parameters are updated by minimizing loss (line 12) and the actor network parameters are updated by policy gradient (line 14). Finally, after all agents updated their actor network and critic network, agents update their target networks by slowly tracking the original networks (line 15).
**Algorithm 1:**O-DCB**Initialization:**  Randomly initialize actor and critic neural network $\mu_{i}\left(s|\theta_{i}^{\mu }\right)$ and $Q_{i}\left(s,a|\theta_{i}^{Q}\right)$ for each agent.  Initialize corresponding target network $Q_{i}^{\prime }$ and $\mu_{i}^{\prime }$ with weights $\theta_{i}^{Q^{\prime }}\leftarrow \theta_{i}^{Q}$, $\theta_{i}^{\mu^{\prime }}\leftarrow \theta_{i}^{\mu }$.  Initialize replay buffer RB.**Algorithm:**  1:   **for** episode in {1,2,3,…}
**do**  2:         Receive initial state $s_{i,1}\leftarrow \left\{0,0\right\}$.  3:         **for**
$t_{j}$ in $\left\{t_{1},t_{2},\ldots \;t_{end}\right\}$
**do**  4:                 for each agent *i*, select $a_{i,j}=\mathrm{Sample}\left[\mu_{i}\left(s_{i,j}\right)\right]$.  5:                 All agents execute actions.  6:                 Calculate reward $r_{i,j}$ according to Equation (10).  7:                 Get new state $s_{i,j+1}$.  8:                 Store $\left(s_{j},a_{j},r_{j},s_{j+1}\right)$ in RB, where $s_{j}=\left(s_{1,j},\ldots ,s_{N,j}\right)$, $a_{j}=\left(a_{1,j},\ldots ,a_{N,j}\right)$, $r_{j}=\left(r_{1,j},\ldots ,r_{N,j}\right)$      and $s_{j+1}=\left(s_{1,j+1},\ldots ,s_{N,j+1}\right)$.  9:                 **for** agent *i* in {1,2,…,N}
**do**10:                       Sample a random minibatch of *L* samples $\left(s_{l},a_{l},r_{l},s_{l+1}\right)$ from RB.11:                       Set $y_{l}=r_{i,l}+\gamma Q_{i}^{\prime }\left(s_{l+1},a_{1,l+1},\ldots ,a_{N,l+1}\right){|}_{a_{k,l+1}=\mu_{k}^{\prime }\left(s_{k,l+1}\right)}$12:                       Update critic by minimizing the loss:
$loss=\frac{1}{L}\sum_{l}{\left(y_{l}-Q_{i}\left(s_{l},a_{l}\right)\right)}^{2}.$13:                       Update actor using the sampled policy gradient:
$\nabla_{\theta_{i}^{\mu }}J\approx \frac{1}{L}\sum_{l}\nabla_{\theta_{i}^{\mu }}\mu \left(s_{i,l}\right)\nabla_{a_{i}}Q_{i}\left(s_{l},\mu_{1}\left(s_{1,l}\right),\ldots ,\mu_{N}\left(s_{N,l}\right)\right).$14:                 **end for**15:                 Update the target networks for each agent *i*:
$$\theta_{i}^{Q^{\prime }}\leftarrow \tau \theta_{i}^{Q}+\left(1-\tau \right)\theta_{i}^{Q^{\prime }},$$
$\theta_{i}^{\mu^{\prime }}\leftarrow \tau \theta_{i}^{\mu }+\left(1-\tau \right)\theta_{i}^{\mu^{\prime }}.$16:         **end for**17: **end for**

### 5.5. Implementation

O-DCB can be implemented in the centralized controller due to limited computation ability and storage space in APs. At the end of each time slot, the centralized controller collects essential information (load size, traffic arrival rate and so on) from each AP. Then, MADDPG is executed. The channel bonding parameters are obtained by the actor neural networks. Finally, new channel bonding parameters are distributed to each AP and APs use the new parameters in the next time slot. Because of the temporal and spatial correlations of traffic load, the learning process will gradually stabilize. Besides, in order to avoid the poor performance in learning, initial learning can be executed in the background by the use of recorded traffic traces.

## 6. Simulation and Performance Evaluation

**Figure 5:**
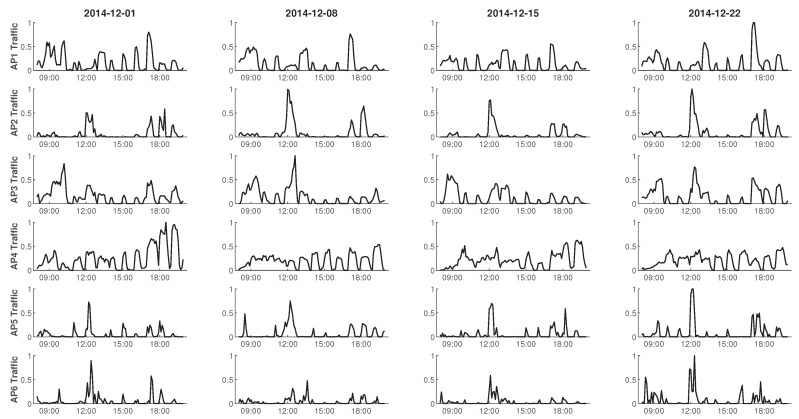
Traffic load changes of AP1–AP6 from 8 a.m. to 8 p.m. in 4 days.

In this section, we present simulation to evaluate the performance of O-DCB. The simulation is performed with TensorFlow 1.13 [45] and Python 3.6. We consider a wireless network where there are 6 APs, and *n*, ac, ax denote the number of 802.11n APs, 802.11ac APs and 802.11ax APs, respectively. The interference relationship is shown in Figure 3. The number of basic channels *K* is equal to 4. We use real traffic traces dataset provided in [17], which are captured from a campus WLAN from September 2014 to January 2015 (The dataset is publicly available at https://github.com/zaxliu/deepnap). Each record in the dataset contains the following information: session ID, user ID, session start time and end time (UTC time), the total number of HTTP requests and bytes requested, etc. Similar with [17], we make the following assumption to mitigate the imperfection in the dataset: we assume a constant packet size $S_{p}$ and uniform packet arrival in each session to translate coarse session-level summary to fine-grained packet arrival process. The traffic load changes of all APs per 6-minutes within 4 days are shown in Figure 5, where the data in each AP are processed with min-max normalization. We only consider traffic load from 8 a.m. to 8 p.m. (UTC+8) in everyday, because there is very little load at night. It is not difficult to see that the repeatability of traffic load fluctuation in a 7-day cycle, which is due to the periodicity of users activities in campus. We can also see that the traffic load is bursty in the daytime, which is a great challenge. In the simulation, the traffic traces of the first three days are used as training set, while the traffic traces in the last day are selected as test set. Feed-forward fully connected neural networks are used as function approximator in MADDPG. The main simulation parameters are listed in Table 1, Table 2 and Table 3.

In particular, independent DQN (IDQN), random selection and fixed configuration are used as benchmark.

- **IDQN.** The simplest approach of learning in multi-agent settings is to use independently learning agents, where each agent independently maximizes its individual reward and treats other agents as part of the environment. This is attempted with *Q*-learning in [46], which is called independent *Q*-learning (IQL). As *Q*-learning can be extended as DQN, IQL can be also upgraded to IDQN easily. In particular, IDQN uses the same state, action and reward with O-DCB. Besides, the hyperparameters of IDQN are also the same with O-DCB, such as the learning rate, the size of replay buffer and minibatch, the structure of DNN, etc.
- **Random selection.** Random selection is very straightforward. In time slot $t_{j}$, for each AP *i*, the channel bonding parameter is randomly selected from its action space $C_{i}$.
- **Fixed configuration.** Fixed Configuration (FC) is always used in real-life WLANs where the channel parameter is fixed for each AP and the widest channel is used.

### 6.1. The Coexistence of 802.11n and 802.11ac

**Figure 6:**
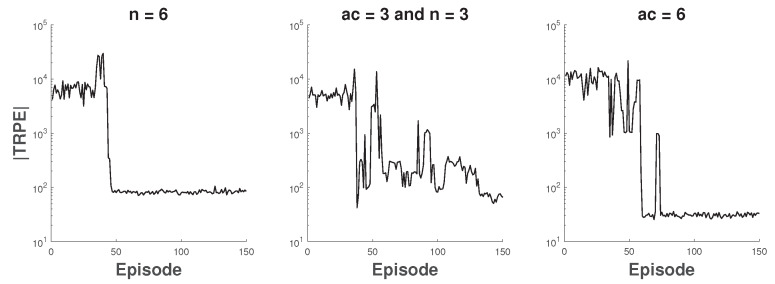
The total reward of each episode during training.

In this part, the coexistence scenario of 802.11n APs and 802.11ac APs is considered, and all APs use DCB. Apparently, 802.11ac APs have larger action space and better channel bonding capability than 802.11n APs. Concretely, we consider three different scenarios as follows: (1) six 802.11n APs; (2) the first three APs are 802.11ac and the rest APs are 802.11n; (3) six 802.11ac APs. In first two scenarios, for each AP, the channel bonding parameters of FC are [(1,2),(3,2),(1,2),(1,2),(3,2),(3,2)] and [(4,4),(1,4),(1,4),(4,2),(1,2),(4,2)] respectively. In the last scenario, the channel bonding parameters of FC are that all APs bond four basic channels. During the training of O-DCB, for each scenario, the absolute value of Total Reward Per Episode (TRPE) is shown in Figure 6. We can see that the training is fluctuant in the second scenario and is relatively stable in other scenarios. During the test, each agent in O-DCB only uses its trained actor network and its current state to generate actions without any extra information. Meanwhile, argmax is used to determine actions from the PMF other than sampling, i.e., $a_{i}=\mathrm{argmax}\;\mu_{i}\left(s_{i}\right)$.

**Figure 7:**
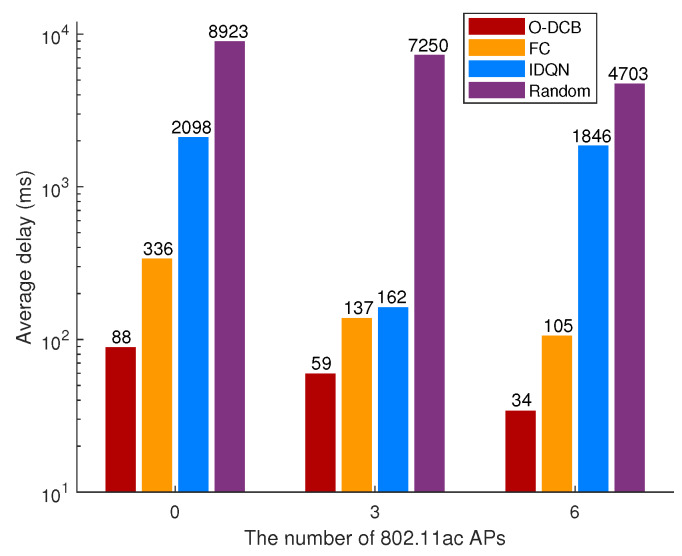
Comparisons of average delay over different algorithms and 802.11ac AP number.

The average delay of total network over different algorithms and 802.11ac AP number is shown in Figure 7. It is easy to see that random selection has the worst performance. IDQN has better performance than random selection but is not as good as FC and O-DCB. This is because IDQN agents are independently updating their policies as learning progresses, which results in non-stationary of the environment from the view of any one agent. Although FC has good performance, there is much room for improvement. After training, O-DCB has shorter delay than FC. For example, in the first scenario, the channel bonding parameters of all APs used most often by O-DCB is [(4,2),(2,2),(4,1),(3,2),(3,2),(2,2)], which proves that using the widest channel is not always optimal. We can also see that the average delay of different algorithms decreases with the increase of 802.11ac APs except IDQN. This is because 802.11ac APs bring better channel bonding capability. However, due to the independent learning and ignoring other agents in IDQN, IDQN is very unstable. For example, when the number of 802.11ax APs is 3, the average delay of IDQN is 162 ms, which is much shorter than other scenarios. As a result, IDQN is unsuitable for the design of on-demand channel bonding algorithm.

### 6.2. The Coexistence of 802.11ac and 802.11ax

**Figure 8:**
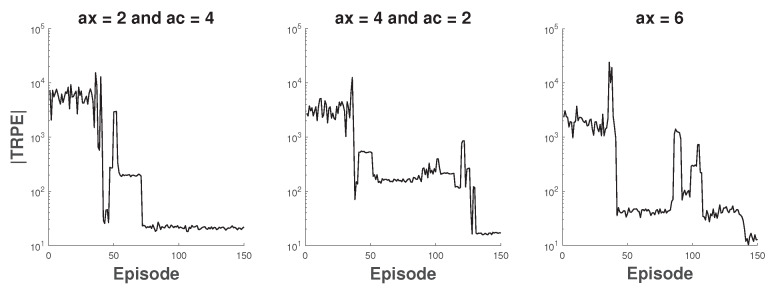
The total reward of each episode during training.

Besides SCB and DCB, 802.11ax can use non-contiguous CA [4]. When not all secondary channels are idle in PIFS, CA can use all idle secondary channels without any contiguity restriction. For example, 802.11ax APs can bond three arbitrary basic channels, which is unachievable in 802.11n/ac. In this part, the coexistence scenario of 802.11ac APs and 802.11ax APs is considered, where 802.11ac APs use DCB and 802.11ax APs use CA. Concretely, we consider three different scenarios as follows: (1) the first two APs are 802.11ax and the rest APs are 802.11ac; (2) the first four APs are 802.11ax and the rest APs are 802.11ac; (3) six 802.11ax APs. In all above scenarios, the channel bonding parameters of FC are that all APs bond four basic channels. The absolute value of TRPE in above scenarios during training is shown in Figure 8.

**Figure 9:**
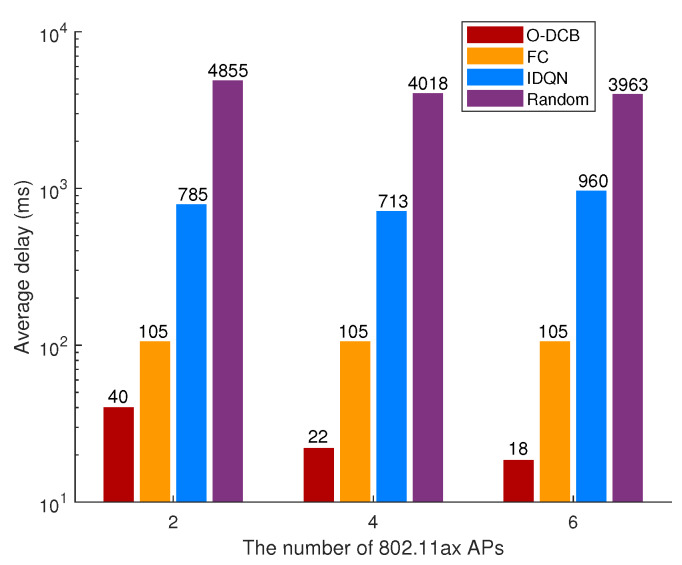
Comparisons of average delay over different algorithms and 802.11ax AP number.

The average delay over different algorithms and 802.11ax AP number is shown in Figure 9. Same as the previous subsection, random selection has the worst performance and O-DCB has the best performance. Similarly, IDQN only has better performance than random selection, which is due to the isolated learning of each agent. The delay of FC is unchanged in three scenarios and is same with the scenario consisting of six 802.11ac APs. This is because CA and DCB will equivalent if all APs bond total channels. Due to the more flexible channel bonding of 802.11ax APs, the average delay of O-DCB is similar or shorter compared with the scenario consisting of six 802.11ac APs, and decreases with the increase of the number of 802.11ax APs.

## 7. Conclusions

In this paper, an on-demand channel bonding algorithm based on MADDPG for heterogeneous WLANs was proposed, where the APs had different channel bonding capability. In O-DCB, the state, action and reward were carefully designed under the consideration of reducing average transmission delay. Real traffic traces collected from a campus WLAN were used to train and test O-DCB. Simulation results showed that O-DCB had good convergence and lower delay than other algorithms.

## Figures and Tables

**Table 1:** Simulation Parameters.

Notation	Definition	Value
N	The number of WLANs	6
K	The number of channels	4
$S_{p}$	Packet size	3 kB
$CW_{min}$	Min. contention window	16
$CW_{max}$	Max. contention window	1024
$R_{B}$	The transmission rate	13, 27, 42.5, 58.5 Mbps
δ	The length of time slot	6 min
η	The weighting factor	−0.25
ϵ	The weighting factor	−1
α	Learning rate	0.001
τ	The tracking parameter	0.01
$S_{RB}$	The size of replay buffer	4096
*L*	The size of minibatch	32
γ	The discount factor	0.95

**Table 2:** Parameters of actor neural network for agent *i*.

Name	Number	Size	Activation Function
Input Layer	1	2	NA
Hidden Layer	2	256,256	ReLU, ReLU
Output Layer	1	8 (802.11n) 12 (802.11ac/ax)	NA

**Table 3:** Parameters of critic neural network for agent *i*.

Name	Number	Size	Activation Function
Input Layer	1	14N−4n	NA
Hidden Layer	2	256,256	ReLU, ReLU
Output Layer	1	1	NA
